# An Observational Study of Patient Characteristics Associated with the Mode of Admission to Acute Stroke Services in North East, England

**DOI:** 10.1371/journal.pone.0076997

**Published:** 2013-10-08

**Authors:** Christopher I. Price, Victoria Rae, Jay Duckett, Ruth Wood, Joanne Gray, Peter McMeekin, Helen Rodgers, Karen Portas, Gary A. Ford

**Affiliations:** 1 Institute for Ageing and Health (Stroke Research Group), Newcastle University, Newcastle Upon Tyne, United Kingdom; 2 Institute of Health and Society, Newcastle University, Newcastle Upon Tyne, United Kingdom; 3 North East Ambulance Service NHS Foundation Trust, Newburn Riverside, Newcastle Upon Tyne, United Kingdom; 4 North of England Cardiovascular Network, Darlington, United Kingdom; Cardiff University, United Kingdom

## Abstract

**Objective:**

Effective provision of urgent stroke care relies upon admission to hospital by emergency ambulance and may involve pre-hospital redirection. The proportion and characteristics of patients who do not arrive by emergency ambulance and their impact on service efficiency is unclear. To assist in the planning of regional stroke services we examined the volume, characteristics and prognosis of patients according to the mode of presentation to local services.

**Study design and setting:**

A prospective regional database of consecutive acute stroke admissions was conducted in North East, England between 01/09/10-30/09/11. Case ascertainment and transport mode were checked against hospital coding and ambulance dispatch databases.

**Results:**

Twelve acute stroke units contributed data for a mean of 10.7 months. 2792/3131 (89%) patients received a diagnosis of stroke within 24 hours of admission: 2002 arrivals by emergency ambulance; 538 by private transport or non-emergency ambulance; 252 unknown mode. Emergency ambulance patients were older (76 vs 69 years), more likely to be from institutional care (10% vs 1%) and experiencing total anterior circulation symptoms (27% vs 6%). Thrombolysis treatment was commoner following emergency admission (11% vs 4%). However patients attending without emergency ambulance had lower inpatient mortality (2% vs 18%), a lower rate of institutionalisation (1% vs 6%) and less need for daily carers (7% vs 16%). 149/155 (96%) of highly dependent patients were admitted by emergency ambulance, but none received thrombolysis.

**Conclusion:**

Presentations of new stroke without emergency ambulance involvement were not unusual but were associated with a better outcome due to younger age, milder neurological impairment and lower levels of pre-stroke dependency. Most patients with a high level of pre-stroke dependency arrived by emergency ambulance but did not receive thrombolysis. It is important to be aware of easily identifiable demographic groups that differ in their potential to gain from different service configurations.

## Introduction

Co-ordinated specialist services reduce the mortality, dependency and economic burden resulting from stroke through a combination of interventions provided by multidisciplinary teams [1,2]. Collaboration between healthcare providers is associated with efficient delivery of urgent care, but the ideal service configuration depends upon local factors including population distribution relative to neuroscience facilities [3]. Pre-hospital redirection of patients by emergency ambulance to a regional acute stroke unit (ASU) consistently provides high quality acute care including thrombolytic therapy but in this configuration other modes of admission reduce service efficiency and hinder meaningful comparison of performance [3-5]. Late presentations are not unusual, often due to milder or atypical symptoms being interpreted by patients as less serious or failing to trigger a pre-hospital redirection response [6-8]. When stroke occurs during hospital admission, care needs are often complex due to co-morbidities which may require treatment outside of a stroke unit setting. Thombolysis may be appropriate for some of these patients, but lengthy transfer to a regional centre for specialist assessment could reduce its effectiveness due to the time dependent nature of treatment [3,9]. Although pre-hospital redirection typically includes all cases of suspected stroke, to improve efficiency some settings have excluded patients who are unlikely to benefit from thrombolytic therapy due to a high level of pre-existing dependency [10-12]. If selective redirection is used, patients admitted to a local hospital would still require access to appropriate high quality multidisciplinary care, and during service planning it is important to ensure that this group are not disadvantaged.

In order to understand the service implications for a region considering a change from local acute stroke care to a centralised hyperacute service, we prospectively examined the volume, characteristics and thrombolysis treatment of consecutive stroke patients who:

1. were transported by emergency ambulance and would have a high likelihood of pre-hospital redirection
2. were not transported by emergency ambulance and would require secondary redirection or local admission
3. did not have a diagnosis made within 24 hours of admission or were already an inpatient for another condition when symptoms started and so would not undergo pre-hospital redirection
4. were transported by emergency ambulance with a high level of pre-existing dependency and would be admitted locally in a selective redirection design.

## Methods

Between 01/09/10-30/09/11, all stroke services within North East, England provided demographic, clinical care process and outcome details of consecutive stroke patients. Twelve ASU served a population of 2.6 million across an area of 3,300 square miles. During data collection there was no planned pre-hospital redirection of suspected stroke patients across organisational boundaries. All services offered intravenous thrombolysis either by direct and/or remote specialist assessment up until 4.5hours after symptom onset [9]. Data from each site was exported monthly to a regional database at the Institute of Health & Society, Newcastle University. Local data protection permission was granted by each NHS organisation. Patient identifiable data was not exported. Ethical approval was not required.

Data were collected prospectively by audit facilitators within local stroke teams. After training, facilitators used structured forms to extract data from clinical records for entry onto the database. If records were unclear, advice was sought from the relevant stroke specialist. A detailed handbook and fortnightly teleconference with the project co-ordinator (VR), database designer (RW) and clinical lead (CP) provided facilitators with additional clarification of clinical definitions and data handling processes. Missing data reports were sent to each site from the central database every month.

Diagnostic status at 24hours after admission was documented for all suspected stroke patients. If the clinical record was unclear or uncertain (e.g. “possible stroke”) facilitators consulted the relevant stroke specialist to make a decision based upon information available in the first 24hours. Facilitators cross-referenced their site data with the hospital coding system weekly to check whether any patients had been discharged with a stroke diagnosis (ICD10 code G46, I61, I62, I63 or I64). If a diagnosis had been made more than 24hrs after admission or on a ward other than the ASU, the facilitator retrieved clinical records and consulted a local specialist to confirm whether this was because of an atypical presentation or new symptom onset whilst already an inpatient for another condition. Patients were only included in the database if a local specialist confirmed a diagnosis of stroke according to the WHO definition, either in person or after review of the clinical records following discharge with a relevant ICD10 code. If at any point after admission patients with suspected stroke received another diagnosis, this was confirmed by a local stroke specialist and they were excluded from the database.

Outcome at discharge was assessed by modified Rankin Score (mRS) [13] and Barthel ADL Index [14]. For patients discharged to a private address the average number of formal (i.e. salaried) daily care episodes was reported. To examine the effect of pre-existing health status on transport mode and outcome, a high dependency group was defined comprising patients with a pre-stroke mRS of 4 or 5. When this information was missing, patients were included if their pre-stroke residence was a licensed nursing home. By this method all admissions were assigned a dependency status.

Data are presented as means (standard deviation) or medians (interquartile range). Statistical comparisons were undertaken using Chi-square test, independent samples t-test and Kruskal Wallis test as appropriate.

## Results

**Figure 1:**
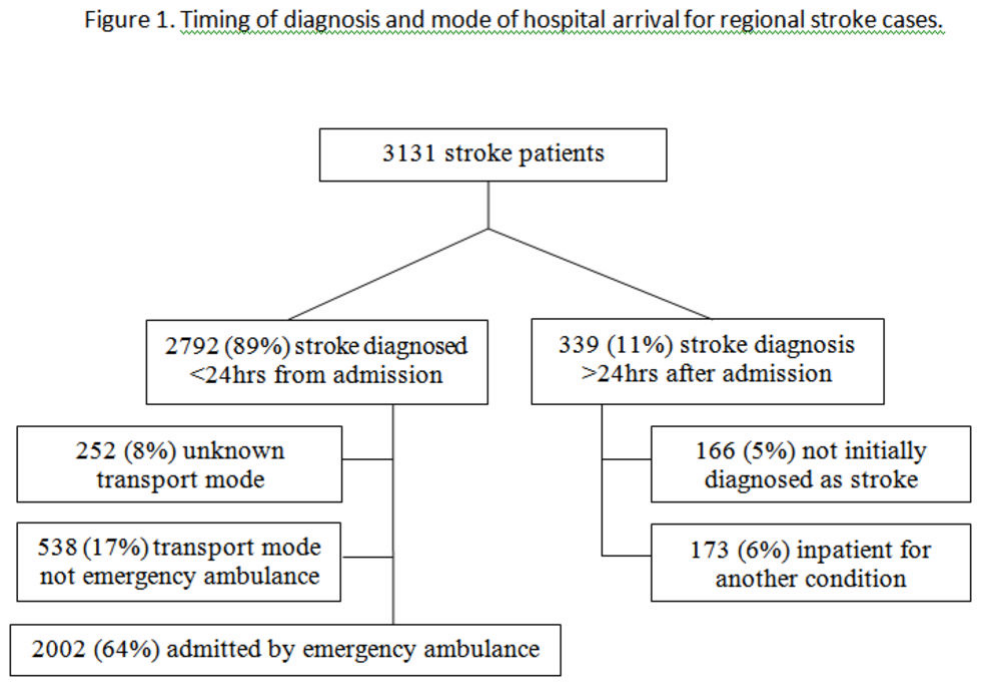
Timing of diagnosis and mode of hospital arrival for regional stroke cases.

Between 01/09/10 and 30/09/11 the mean number of consecutive months contributing data by regional ASUs was 10.7 (range: 7-12). After correction by the time interval that each site contributed data, we estimated from hospital episode statistics that the regional database should contain 3571 cases with a primary diagnosis of stroke. The regional ASUs recorded details of 3131 patients with a new diagnosis of stroke (88% of estimated possible cases). Figure 1 shows their diagnostic and transportation status.

Overall, 339/3131 (11%) cases did not have a diagnosis of stroke made within 24 hours of their hospital admission: 166 (5%) were atypical presentations according to local specialist judgement and 173 (6%) were already an inpatient for another condition. After exclusion of 252 admissions with an unknown transport mode, 877/2879 (30%) of the cohort were either inpatients at the time of stroke diagnosis or did not arrive by emergency ambulance. When a stroke diagnosis was made in the first 24 hours, 2002/2540 (79%) patients were known to have arrived by emergency ambulance. Table 1 compares their characteristics to the 538 new stroke admissions who arrived by low priority ambulance dispatch following primary care review (n=159) or self-presentation (n=379). New admissions who did not attend via emergency ambulance were younger, less dependent, and less likely to have a severe clinical stroke subtype. There was a trend towards an association with male gender and ischaemic aetiology. Emergency ambulance patients were more likely to have a previous history of stroke or TIA and a known symptom onset time. They arrived in hospital an average of 9 hours sooner. There was no difference in presentations according to day of the week.

**Table 1:** Characteristics of patients with a stroke diagnosis confirmed within 24 hours of admission.

	Emergency ambulance (n=2002)	Other mode of admission (n=538)	p value
Male gender (%) [missing]	925 (46%) [3]	299 (56%) [0]	0.08
Age mean (SD) [missing]	75.7 (12) [19]	69.3 (13) [32]	<0.01
Pre-stroke mRS median (IQR) [missing]	0 (0-2) [465]	0 (0-1) [139]	<0.01
Pre-stroke mRS 0-2 (%) [missing]	1287 (84%) [465]	374 (94%) [139]	<0.01
Pre-stroke care home resident (%) [missing]	169 (10%) [277]	6 (1%) [64]	<0.01
Previous history of stroke or TIA (%) [missing]	587 (31%) [105]	101 (22%) [82]	<0.01
Lacunar stroke (LACS) (%)	496 (25%)	178 (33%)	<0.01
Partial anterior circulation stroke (PACS) (%)	561 (28%)	171 (32%)	0.08
Total anterior circulation stroke (TACS) (%)	548 (27%)	30 (6%)	<0.01
Posterior circulation stroke (POCS) (%)	177 (9%)	101 (19%)	<0.01
Undetermined clinical classification (%)	223 (11%)	58 (11%)	0.46
Ischaemic stroke (%) [missing]	1734 (89%) [63]	491 (96%) [26]	0.10
Known onset time (%)	1333 (67%)	287 (53%)	<0.01
Onset –admission hours median (IQR)	1 (1-3)	6 (2-17)	<0.01
Onset –admission hours mean (SD)	5.3 (25)	14.5 (27)	<0.01
Admitted at a weekend (%)	515 (26%)	124 (23%)	0.43

Intravenous thrombolysis was received by 246/2540 (10%) of admissions when transport mode was known (Table 2). 56/246 (23%) involved a remote specialist decision. The documentation of thrombolysis assessments was not related to transport mode. Treatment occurred less often when arrival did not involve an emergency ambulance, reflecting the later and milder presentations, but 23/538 (4%) still received treatment comprising 20 self-presentations, 2 non-urgent ambulance transfers following primary care review in the community and 1 transfer of a patient after initial non-urgent admission to a neighbouring hospital site within the same organisation. Median door to needle time was longer following emergency ambulance transport but this was not a significant difference. Intravenous thrombolysis was received by an additional 13/339 patients who were already an inpatient when their stroke diagnosis was made.

**Table 2:** Characteristics of intravenous thrombolysis provision.

	Emergency ambulance (n=2002)	Other mode of admission (n=538)	p-value
Thrombolysis assessments documented (%)	594 (30%)	159 (30%)	0.47
Thrombolysis treatments (%)	223 (11%)	23 (4%)	<0.01
Treated group age median (IQR)	74 (67-80)	72 (66-77)	0.23
Treated group pre-stroke mRS median (IQR)	0 (0-2)	0 (0-1)	0.55
Door to needle minutes median (IQR)	75 (51-100)	63 (38-90)	0.39

The overall inpatient mortality for patients diagnosed with stroke <24 hours of admission was 338/2540 (13%). 84/1633 (5%) of survivors who had been admitted from a private residence were discharged to a care home. Table 3 shows that outcome varied according to mode of admission. The younger age and milder stroke severity of patients who did not attend by emergency ambulance is reflected in their lower mortality, dependency and care requirements.

**Table 3:** Patient outcomes at discharge.

	Emergency ambulance	Other mode of admission	p value
Inpatient mortality (%)	328 (18%)	10 (2%)	<0.01
Discharge mRS median (IQR) [missing]	2 (1-4) [547]	1 (0-2) [191]	<0.01
Discharge mRS 0-2 [missing]	630 (56%) [547]	295 (85%) [191]	<0.01
Discharge Barthel median (IQR) [missing]	17 (9-20) [870]	20 (18-20) [306]	<0.01
Discharges to any private residence (%)	1121 (67%)	428 (81%)	<0.01
Discharges to residential home (%)	71 (4%)	2 (<1%)	<0.01
Discharges to nursing home (%)	135 (8%)	8 (2%)	<0.01
Discharges to other destination e.g. respite (%)	212 (13%)	64 (12%)	0.57
Discharge destination unknown (%)	135 (8%)	26 (5%)	0.20
Patients with daily care visits at home (%) [missing]	165 (16%) [98]	20 (6%) [74]	<0.01
New discharge to a care home (%) [missing]	80 (7%) [32]	4 (1%) [18]	<0.01

Patients with high dependency prior to stroke accounted for 155/2792 (6%) admissions who had a diagnosis made within 24 hours. None received intravenous thrombolysis. Six (4%) were admitted by non-urgent ambulance following a primary care review and none attended by private transport. The remaining 149 accounted for 7% of emergency ambulance admissions. This group was predominantly female and an average of 9 years older compared to 1853 less dependent cases (Table 4). A greater proportion presented with the most severe stroke category (43% versus 26% TACS) and a known onset time was less common (55% versus 68%). Mortality was nearly twice that of patients living independently outside of nursing care before stroke onset.

**Table 4:** Emergency ambulance patient characteristics according to pre-stroke dependency.

	High dependency (n=149)	Not high dependency (n=1853)	p value
Pre-stroke mRS median (IQR) [missing]	4 (4-4) [24]	0 (0-1) [441]	<0.01
Care home resident pre-stroke (%) [missing]	99 (67%) [10]	70 (4%) [267]	<0.01
Age mean (sd) [missing]	84 (9) [0]	75 (12) [19]	<0.01
Male gender (%) [missing]	42 (28%) [0]	883 (48%) [3]	<0.01
Previous history of stroke or TIA (%) [missing]	73 (49%) [4]	541 (28%) [101]	<0.01
Lacunar stroke (LACS) (%)	21 (14%)	472 (26%)	<0.01
Partial anterior circulation stroke (PACS) (%)	37 (25%)	524 (28%)	0.22
Total anterior circulation stroke (TACS) (%)	64 (43%)	484 (26%)	<0.01
Posterior circulation stroke (POCS) (%)	5 (3%)	172 (9%)	<0.01
Undetermined clinical classification (%)	22 (15%)	201 (11%)	0.38
Ischaemic stroke (%) [missing]	126 (85%) [6]	1608 (87%) [57]	0.45
Known onset time (%)	82 (55%)	1251 (68%)	0.02
Onset –admission hours median (IQR) [missing]	1 (1-2) [68]	1 (1-4) [610]	<0.01
Inpatient mortality (%)	41 (28%)	287 (16%)	<0.01

## Discussion

We have described the mode of admission and demographic characteristics of stroke patients from twelve ASU in order to inform the configuration of regional acute stroke services. Primary redirection by emergency ambulance to a regional unit would be feasible for the majority of patients. However 30% did not present by emergency ambulance or had a diagnosis of stroke made more than 24hrs after local admission and would have less chance of rapid admission to a central ASU. Non-urgent ambulance admissions (5% of total cohort) could still undergo pre-hospital redirection with appropriate protocols in place, but the remaining 25% would require secondary transfer (i.e. after local medical assessment) because of arrival by private transport or diagnosis after admission / inpatient symptom onset. As non-emergency admissions tend to have less severe stroke and therefore better outcomes than emergency ambulance patients, there could be less health gain from this additional transfer process compared to primary redirection by emergency ambulance. Therefore services could consider local sub-acute care provision on an individual basis after discussion with the regional centre, assuming that the local facility could respond adequately to the same care quality standards including rapid initiation of secondary prevention.

Although attendance without ambulance involvement occurred frequently enough to require consideration in the future planning of services, this proportion was smaller than reported elsewhere. In the United States, the 2003 National Hospital Ambulatory Medical Care Survey (NHAMCS) observed that for 630,402 stroke patients evaluated in emergency departments the mode of arrival was 53% by ambulance, 43% private transport and 4% other/unknown [15]. These proportions did not change significantly between 1997 and 2008 [16]. Across 195 hospitals in Georgia, Illinois, Massachusetts, and North Carolina, USA during 2005–2007, 48% of 56,969 patients were transported by emergency ambulance directly from the scene, 11% were transferred from another hospital and 39% used other transport [4]. A ten site audit in Michigan, USA reported that 59% of admissions were by ambulance, also finding that this mode was used more often by women than men even after correction for age differences [17]. This demonstrates the importance of obtaining data which is directly relevant to the region where services are under review. Undefined complex healthcare provider interactions, data capture processes, behavioural and financial influences upon utilisation of services in other settings may lead to incorrect assumptions about their efficiency and the resources required [18].

There is general agreement that mode of admission is linked to outcome through demographic factors and stroke severity. In NHAMCS, ambulance patients were older and more likely to be admitted to an intensive care unit [15]. In Georgia USA, the 172/409 patients who arrived within 2 hours of symptom onset were more likely to have attended by ambulance with a greater severity of neurological deficit and inpatient mortality [19]. A prospective study across 14 tertiary hospitals in Korea showed significant associations between arrival by emergency ambulance (36% of total admissions), stroke severity, previous stroke and poorer outcome [20]. Therefore it was not surprising that we observed a higher mortality and institutionalisation rate for emergency ambulance patients. For those discharged home a larger proportion required daily care visits.

With a centralised model of acute stroke care the small number of non-emergency admissions (predominantly self-presentations) and inpatient stroke cases who received thrombolysis would be at risk of losing this opportunity, but treatment could still be provided through remote assessment followed by “drip and ship” transfer [21]. Telemedicine can provide effective local assessment for intravenous thrombolysis [22], but selected patients may require transfer for neurosurgical assessment or if evidence accumulates to support other acute treatments only available at a neuroscience centre.

In practice it is also necessary to consider how the performance of pre-hospital patient identification affects the efficiency of redirection. In the UK, the Face Arm Speech Test (FAST) is the standard tool for stroke symptom recognition and identifies approximately 80% of cases [23]. Like non-emergency admissions, stroke patients who are FAST false negative could be admitted to a centralised ASU by secondary redirection, but are more likely to miss thrombolysis treatment if remote specialist assessment is unavailable. In addition, patients with a stroke mimic condition resulting in a FAST false positive status would be taken to the regional ASU. When planning the resources needed to support a redirection service, it would be important to recognise these subgroups, particularly regarding delivery of time-dependent treatments and the central accumulation of patients with stroke mimic conditions, some of whom may be too unwell for early repatriation to their local hospital. In Calgary [5], 29% of referrals from local hospitals and paramedics to a centralised regional service did not have a stroke or TIA despite the use of a pre-hospital screening tool [11]. Across nine hubs in Orange, County, California between April 2009 and April 2010, 443/1360 (32.6%) of emergency ambulance transported patients with suspected stroke were given a non-stroke diagnosis [24]. Organisational transfer agreements, feedback and constant re-enforcement of protocols though training is required to minimise the impact of inappropriate displacement for patients and services.

In 2010 the National Sentinel Stroke Audit for England, Wales and Northern Ireland reported an overall 30-day mortality of 17% [25]. In our regional cohort, emergency ambulance admissions identified as “high dependency” had a far higher mortality. This is not surprising in view of their co-morbidities, greater age and stroke severity, which are strongly associated with a poor outcome in predictive algorithms [26,27], but pre-stroke disability alone [28] has been identified as a predictor of mortality and length of stay. None of this group received thrombolysis. If the region adopted a selective pre-hospital protocol excluding patients with a high level of pre-stroke dependency from redirection for thrombolytic therapy [10-12], 7% emergency ambulance admissions would require local ASU care.

A national mass media campaign for stroke symptom awareness was active during the period of data collection and so the proportion of self-presentations to A&E or via primary care reflects contemporary patterns of behaviour. There is debate about the long term effectiveness of attempts to raise public awareness for unpredictable conditions which require urgent action [7,25,29-32]. It is interesting to note that patients with a previous history of stroke or TIA were better represented in the emergency ambulance group and residents from care home settings nearly all attended by emergency ambulance, presumably due to the constant presence of healthcare workers. Previous experience of symptoms by patients, their families and professional carers may be a more powerful factor than abstract health knowledge. Ten years previously the proportion of patients attending by emergency ambulance accounted for only 51% of stroke admissions to one unit within the same region [23]. The increase in urgent response is likely to be multi-factorial including public and professional awareness, but also enforcement of formal ambulance protocols and more general influences such as contractual agreements for primary care cover out of hours. Despite these changes it is likely that a proportion of milder patients will always present late to primary care or directly to emergency departments.

The late or non-arrival of up to 30% of patients at a regional stroke centre may also hinder the comparison of care between services. Data sharing between central and associated local sites is needed to ensure that all patients feature in the denominator of care quality metrics [3]. If there is incomplete data capture then the better prognosis of non-emergency patients might favour local reporting of outcomes, whereas the late and less typical presentations of patients who are not redirected might favour central reporting of acute care quality indicators such as thrombolysis rates and stroke unit access. Therefore we recommend that services specify the proportion of admissions by emergency ambulance or separately provide the summary data for emergency and non-emergency admissions so that comparison can be made of equivalent groups between services with different configurations.

With the resources available it was challenging to capture complete data for all stroke admissions across multiple sites. Establishing the database within organisational computer systems and the appointment of audit facilitators created delays which prevented data collection for a full 12 months at some sites. According to hospital episode statistics, approximately 12% of stroke cases were not identified. As facilitators were based on the ASU it seems unlikely that the missing data are biased against ASU admissions and are more likely to reflect patients who did not follow a typical clinical pathway. Missing data also limits the certainty of conclusions about the care process and outcomes for known cases. Consistent with the national audit process for acute stroke care [25], information collection depended upon self-report by clinical teams and a much greater resource would have been required to employ independent clinicians to objectively confirm data whilst blinded to each ASU site. We used the local specialist opinion that 5% of presentations could not have been attributed to stroke within 24 hours of admission, but it is possible that some could have been identified earlier in other settings particularly through the use of additional brain imaging techniques. However from an operational viewpoint these patients would be less likely to undergo pre-hospital redirection.

## Conclusions

During the planning of ASU provision it is important to recognise patient characteristics, behaviours and diagnostic difficulties which may impact upon service efficiency. In this region the majority of patients with a new diagnosis of stroke were admitted by emergency ambulance. Due to easily identifiable differences in population characteristics such as stroke severity and age, outcomes varied according to the mode of admission and previous level of dependency. It is important that the contribution towards overall service performance is transparent for all subgroups so that the quality of care and outcomes can be reported for the whole population.
